# Research following trauma in minority ethnic and faith communities: lessons from a study of the psychosocial sequelae of the Christchurch mosque terror attacks

**DOI:** 10.1192/bjo.2023.641

**Published:** 2024-01-11

**Authors:** Ruqayya Sulaiman-Hill, Richard Porter, Philip Schluter, Ben Beaglehole, Shaystah Dean, Sandila Tanveer, Joseph Boden, Caroline Bell

**Affiliations:** Department of Psychological Medicine, University of Otago Christchurch, New Zealand; Te Kaupeka Oranga – Faculty of Health, Te Whare Wānanga o Waitaha – University of Canterbury, New Zealand; and Primary Care Clinical Unit, School of Clinical Medicine, The University of Queensland, Australia

**Keywords:** Trauma and stressor-related disorders, stigma and discrimination, terrorist attack, ethnic diversity, Muslim

## Abstract

Recruiting participants for research from highly traumatised ethnic and faith communities requires a participatory and trauma-informed approach that considers logistic barriers, as well as trauma-related and culture-specific issues. Active community engagement through every stage of the project and employing community members in research roles can help build trust, identify and mitigate concerns early, prevent re-traumatization, and ensure that findings will be of value to the community. Some of these research challenges are discussed in the context of the Christchurch mosque terror attacks. These insights may be helpful for researchers and clinicians working in similarly challenging environments.

Conducting research with minority groups in the wake of a highly traumatic incident, such as a terrorist attack, presents significant challenges, especially when a substantial portion of a community is affected. Inherent difficulties in minority group research, including cultural and linguistic differences, stigma, confidentiality concerns, literacy issues and a potentially rudimentary understanding of research processes, are further compounded by exposure to the traumatic event. These challenges are intensified when a community is targeted and vulnerable because of visible differences. To navigate these obstacles, a participatory and trauma-informed approach is essential, addressing logistical barriers, trauma-related issues and culture-specific factors to ensure the ethical integrity and success of the research. By exploring these challenges in the context of a terrorist attack specifically directed at a minority faith community, we offer insights into some of the unique considerations and approaches required in such circumstances.

## Context

### The Christchurch mosque terrorist attacks

On 15 March 2019, a White supremacist gunman attacked Muslim worshippers during Friday prayers at two mosques in Christchurch, New Zealand. This attack is one of the worst mass shootings in recent years and is unprecedented in modern New Zealand,^1^ where the threat of terrorism is generally considered low.^2^ It resulted in 51 fatalities, with a further 40 individuals sustaining serious gunshot wounds, and in some cases, life-changing injuries. At least another 250 survivors witnessed the atrocities. Simultaneous live-streaming and recording also resulted in widespread and repeated exposure and traumatisation.

### Community trauma exposure characteristics

The Christchurch Muslim community is small (approximately 4000 people) and very diverse, with more than 40 ethnicities represented. Many are migrants or former refugees from regions where pre-existing trauma exposure from conflict or terrorism is common. Social connections are strong and cross ethnic lines. Virtually everyone was affected by physical or emotional proximity to the attack,^3^ with most people meeting criteria for trauma exposure through direct experience of the terrorist act, being a witness, learning that it happened to close family or friends, or repeated or extreme exposure to details of the incident.^4^ A significant number of people had multiple and overlapping exposures. This included survivors, family members, those who were bereaved, injured survivors and people who suffered combinations of these experiences. Although terms like ‘victim’ are often used by the media or in the justice system, it is important to note that many people do not like to be labelled as victims. In this community, their preferred terminology is ‘survivor’ for those present, ‘witness’ for those nearby and ‘*shuhada*’ (martyr) for those who died. Although fear and heightened anticipation are inherent characteristics of terrorist attacks,^5^ the focus on faith in this case exacerbates these distressing features, especially for Muslims, who are a vulnerable and visible minority group. This targeted experience not only perpetuates ongoing fears, but also adds additional layers of stress, potentially creating trust issues with authorities. Moreover, beyond individual effects, the targeting of Muslims in this act of terrorism intensifies collective trauma within the community, something likely to be especially profound for groups with collectivist values^6^ – a common characteristic within Muslim communities.

### The March 15 project

The March 15 research project, a collaboration between two Christchurch universities, the regional public health service and the affected community, was designed to assess the psychosocial consequences of the terrorist attack on community members. Aligned with the Substance Abuse and Mental Health Services Administration (SAMHSA) guidance ‘Concept of Trauma and Guidance for a Trauma-Informed Approach’,^7^ our research aimed to understand the effects of trauma while at the same time preventing re-traumatisation. We emphasised the importance of trauma screening and assessment, providing direct benefits for participants by assessing clinical needs and benefitting the wider community through evidence-based recommendations for funding and planning of support services.^8^ As recommended, community voices were integral to our approach, which we achieved by employing Muslims in research governance and leadership positions, as well as including individuals from all exposure groups in advisory roles.

The project utilised a longitudinal mixed-methods format, incorporating self-report measures, a diagnostic clinical interview and a qualitative substudy.^8^ Applying the trauma-informed approach,^7^ we actively avoided potentially triggering questions, used experienced mental health clinicians to conduct clinical interviews and ensured that access to support services was available.

Rather than focusing solely on the adverse effects of the shootings, we also highlighted positive outcomes such as post-traumatic growth and well-being. Recognising the centrality of religious perspectives on suffering and trauma for this population, we also included a religious coping measure. In addition, participants were given the opportunity to take part in a qualitative study to further explore these key components.^9^

### Ethics and consent

The authors assert that all procedures contributing to this work comply with the ethical standards of the relevant national and institutional committees on human experimentation and with the Helsinki Declaration of 1975, as revised in 2008. All procedures involving human patients were approved by the New Zealand Health and Disability Ethics Committee (reference 19/NTA/147). All participants provided informed consent, either written or online via REDCap software for Windows, v9.1.0 (Vanderbilt University, Nashville, TN, USA, hosted for this study by the University of Otago, New Zealand; see https://projectredcap.org/software). This confirms that any participant has consented to the inclusion of material pertaining to themselves, that they acknowledge that they cannot be identified via the manuscript and that the participant has been fully anonymised by the author. The study is registered with the Australian New Zealand Clinical Trials Registry (identifier ACTRN12620000909921).

## Lessons learned

### Working with the affected community

We adopted a participatory methodology because we believe that this research needed to prioritise the health and well-being of the affected community by collaborating with the groups of people who were the focus of the research.^10^ Active engagement with the community throughout every phase of the process also helped us to address practical challenges as they arose.^11^ Research outcomes were agreed upon that not only provided direct clinical benefits, but also aimed to establish an evidence base for other communities that might be affected in the future. The community advisory board, which comprises 18 representatives from all affected groups, continues to be instrumental in this process. An additional benefit was that employing community members in various research positions enabled us to recognise and use existing strengths and resources within the Muslim community. This played a crucial role in building trust, identifying community concerns and yielding meaningful research findings that resonated with affected groups. Involving local Muslim community members in key research roles also provided legitimacy for the project and, crucially, played an important role in strengthening trust, especially through their leadership contributions and established reputation. Three members of the research leadership team, including a principal investigator, are Muslim, and almost 30 research assistants were employed as recruiters, interviewers/interpreters and translators in various phases of the project. They were a crucial link to understanding the cultural diversity and dynamics of the various ethnic identities and social structures, as in some cases, this would inform the need for different approaches.^12^ All research assistants were appropriately qualified and selected based on their professional expertise, ethnicity, diverse language repertoire and interpersonal relationships,^13^ and we provided them with training, ongoing supervision and support.

### Promoting participation

As no formal sampling frame or comprehensive victim list was available, it was essential for us to utilise the social networks of our Muslim researchers. To maximise exposure, we employed multilingual advertising using both print and online methods, and we actively encouraged individuals to share information widely via their social media networks. We prepared translated project materials to reflect the linguistic composition of the community, and interpreter support was available.^8^ In an effort to minimise practical barriers,^14^ we encouraged flexibility in scheduling and interview locations, including conducting interviews in people's homes if that was their preference. We also offered childcare assistance and accommodated work and school commitments, a gesture that was well received, especially by directly affected participants who had limited resources and family support.

After terrorist attacks and other disasters, responders, health providers and other carers often focus their attention on bereaved families and injured individuals, unintentionally neglecting other survivors and the broader community. This may create perceptions within communities, particularly for people who are uninjured, that there are hierarchies of impact and need.^15^ In this context, it is important to note that despite considerable exposure to the incident, both directly and vicariously, some individuals did not categorise themselves as victims, and felt that their perspectives would not be relevant. This tendency was in part driven by an inclination to downplay their own suffering because of the relative nature of their exposures, a dynamic exacerbated by the predominant focus on victims in the legal process and police records. Recognising these complexities, we aimed to address this issue by highlighting the broader community benefits of the research, ensuring that their voices were also heard.

Terror-related experiences can significantly influence the willingness of survivors to participate in research, especially for those people reluctant to be reminded of the incident.^16^ During the initial stages, research participation may not be a priority for some, although others may feel compelled to contribute out of a sense of civic responsibility.^16^ For survivors engaged with the justice process or in the media spotlight, interview fatigue arising from the repeated sharing of their narratives poses another potential barrier. This is particularly relevant within the framework of extended legal processes, as evidenced by the coronial inquiry unfolding in Christchurch 4.5 years after the attack, which has resulted in significant re-traumatisation across the community. The feedback provided by the advisory board highlighted similar challenges and significant dates that would likely affect the research, which allowed us to make appropriate adjustments to interview schedules at those times.

### Stigma

The proposed research received substantial support within the community from the outset, as many people recognised the inevitability that there would be long-term psychosocial consequences following such profound trauma. However, Muslims are often uncomfortable discussing mental health concerns or seeking support because of stigma, so such openness to talk about these topics was unexpected. Nevertheless, some reluctance to participate emerged, primarily stemming from concerns regarding familiar individuals in the research team and the potential implications for confidentiality. To address this, we deliberately maintained a separation between the Muslim recruitment and non-Muslim clinical teams. Participants were identified only by codes, ensuring that research assistants, aside from those involved in specific participant recruitment, remained unaware of participants’ identities. In addition, none of the Muslim team members had access to data about diagnoses or treatment referrals.

However, although also providing non-Muslim staff as an option and placing a strong emphasis on confidentiality, the challenge of stigma persisted. This often took the form of label avoidance or stigma by association, where individuals actively avoided diagnostic labels because of anticipated negative consequences.^16^ Concerns primarily related to potential implications for immigration status, social interactions or the impact on children if a family member were diagnosed with a psychiatric disorder. As recruitment slowed after the initial weeks, it became apparent that mitigating potential stigma concerns would be crucial to ensure broader community acceptance.

To address this challenge, we intentionally shifted our research promotion approach away from a narrow focus on mental health effects, but maintained the same fundamental research aims. The study was rebranded as a well-being project, placing greater emphasis on community-wide benefits, such as helpfulness of services, in the advertising. This not only aimed to counteract the perception that our study was exclusively focused on mental health effects, which we feared could discourage potential participants because of concerns regarding stigma, but by highlighting issues of shared concern, such as financial, immigration or other secondary stressors, we hoped to project a more inclusive and culturally acceptable message where individuals would feel more at ease and, in turn, be more inclined to participate. The project motto, ‘Help us, help you, help our community’, was designed to resonate with prospective participants who saw themselves as contributors to community well-being rather than individuals in need of assistance.

Simultaneously, as recruitment from the most affected groups slowed, we leveraged the additional capacity to extend participation to members of the wider Christchurch Muslim community who were in the city during the attack. It is important to highlight that our core research objectives were unchanged, and recognising the pervasive experience of collective trauma throughout the entire community, a consistent trauma-informed approach was applied to all participants.

### Trust and understanding of the research process

Despite providing multilingual resources, some people still had limited understanding of psychological or clinical research, or appreciation of the potential wider benefits. Concerns about confidentiality breaches leading to retribution, especially if personal information falls into the hands of outsiders, have been reported in medical and psychiatric settings elsewhere.^17^ This may be a particular concern for former refugees and others from regions where mistrust of research or government institutions is common. Additionally, a lack of resonance with Western approaches to mental health and unfamiliarity with healthcare systems can pose challenges, especially when there is suspicion of the very ethical processes designed to protect their interests. Researchers must be mindful of historical human rights violations or traumas that communities may have faced, and factor this into their work to minimise potential harm. Establishing trust in the researchers and their intentions is therefore a crucial component.

### Dual relationships

Involving local Muslims, who held both professional expertise and personal investment in the project's success, not only addressed access challenges, but also helped to build Muslim workforce capacity as a positive outcome.^18^ Individuals from the community, in contrast to external researchers, have proven more effective in improving health outcomes in settings characterised by collective stress experiences elsewhere.^6^ This emphasises the important role of Muslim research assistants as essential contributors to the well-being of their communities, by playing a critical role in promoting research participation and facilitating pathways to therapeutic interventions, if necessary.

However, from a researcher's perspective, challenges concerning dual relationships also emerged for some Muslim staff. Dual relationships occur when individuals work with someone with whom they already have a pre-existing relationship, whether it is an individual or a group. For our research staff, this mostly manifested as some participants not being aware of or not recognising their professional backgrounds or qualifications, prompting questions about their roles and involvement. The complexities surrounding dual relationships, vicarious traumatisation, difficulties setting professional boundaries and the need for ongoing supervision for Muslim staff engaged in various roles following the mosque terror attacks are the central focus of another ongoing qualitative study.

## Recommendations

There is a paucity of evidence on safe and effective individual- and community-level strategies to improve mental health after terrorist incidents affecting minority groups.^19^ Research with non-Western communities with collectivist values and collective trauma histories is limited,^6^ as is data relating to research participation following acts of terrorism.^16^ In light of the challenges we experienced during community engagement in the aftermath of the Christchurch terrorist attack, we make the following recommendations:

- Prioritise the well-being of trauma-affected communities by adopting participatory methodologies. Collaborate with those groups that are the focus of the research to leverage existing strengths and resources. Acknowledge the diversity of effects and ensure the research focus is not solely on the bereaved and injured.
- Engage a community advisory board with representatives from all affected groups. This builds trust and legitimacy for the project and ensures that the opinions of all groups are heard.
- Ensure cultural and linguistic representation in the research team, to understand the dynamics of various ethnic identities and social structures within the affected community, improve connections with participants and tailor research materials accordingly.
- Promote participation by using social networks within the community. Utilise multilingual advertising and flexible interview options to reduce practical barriers.
- Implement a flexible, trauma-informed approach and acknowledge variability in individuals’ readiness to participate. Also, consider the effects of stigma, and endeavour to mitigate its effects by emphasising confidentiality and highlighting the broader community benefits of the research.
- Understand the effect of dual relationships encountered by staff when working within their communities, and ensure ongoing supervision and support for them.

## Data Availability

Data availability is not applicable to this article as no new data were created or analysed in this study.
